# Supplementary material for: Improved maximum growth rate prediction from microbial genomes by integrating phylogenetic information
Source: Nat Commun. 2025 May 7;16:4256. doi: 10.1038/s41467-025-59558-9 (PMC12059116; doi:10.1038/s41467-025-59558-9)
Supplement: Supplementary file 1 — Supplementary Information [file 41467_2025_59558_MOESM1_ESM.pdf]

Supplementary information

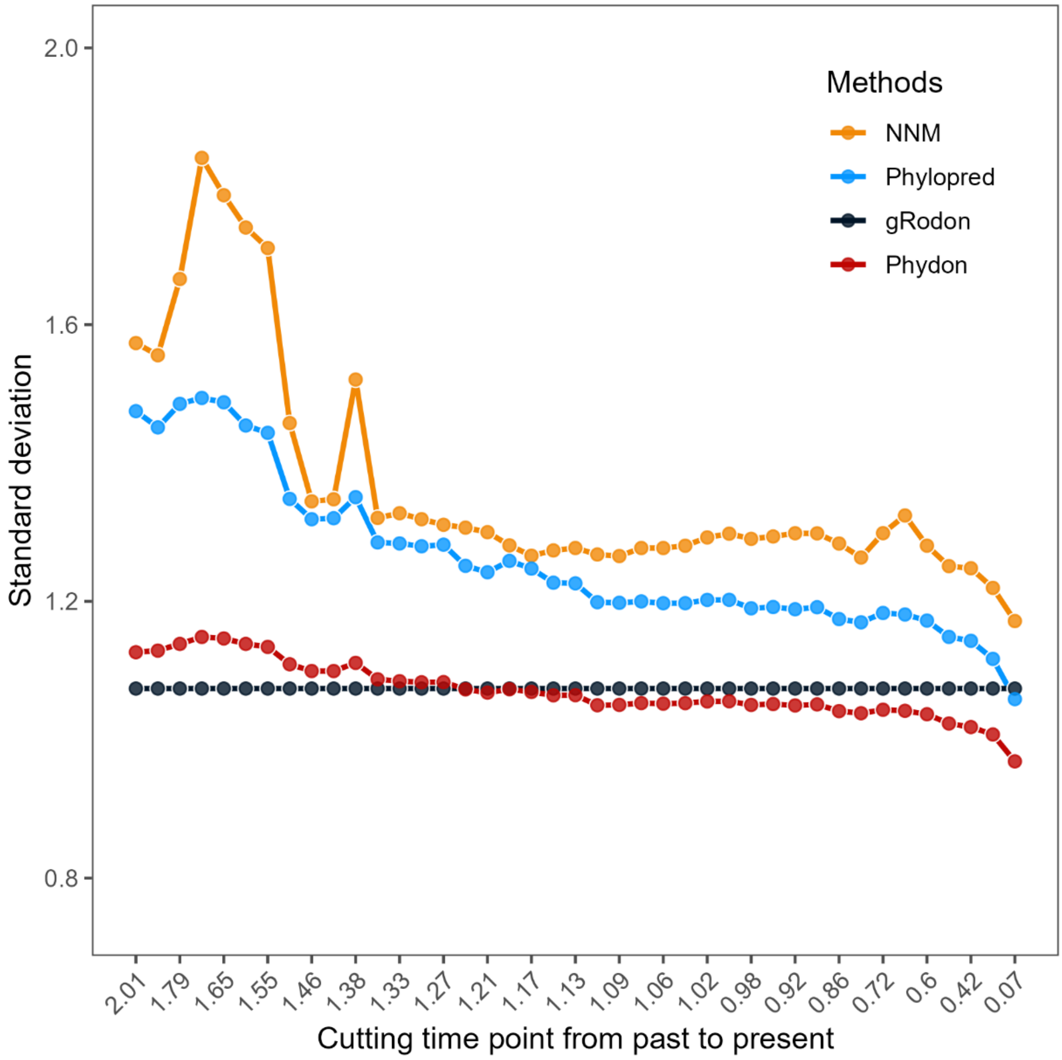

Figure S1. The standard deviation of the prediction error from the four prediction methods varies with the cutting time.

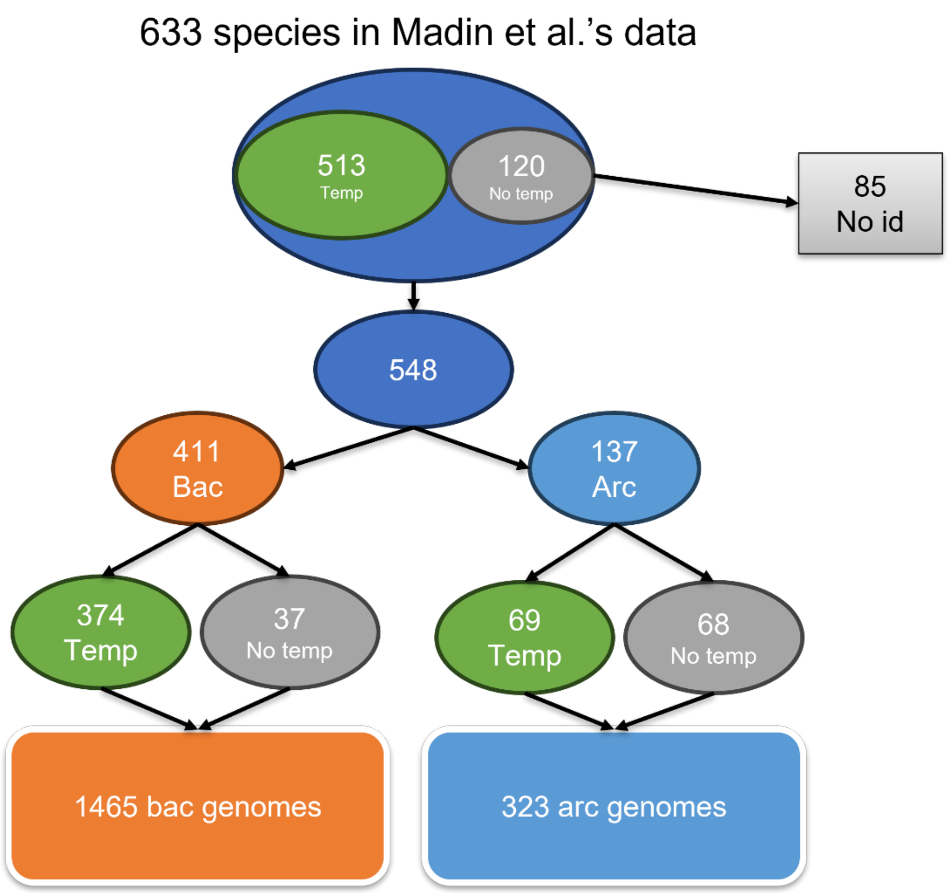

Figure S2. The description of Madin et al **[1]**.'s data used in this study.

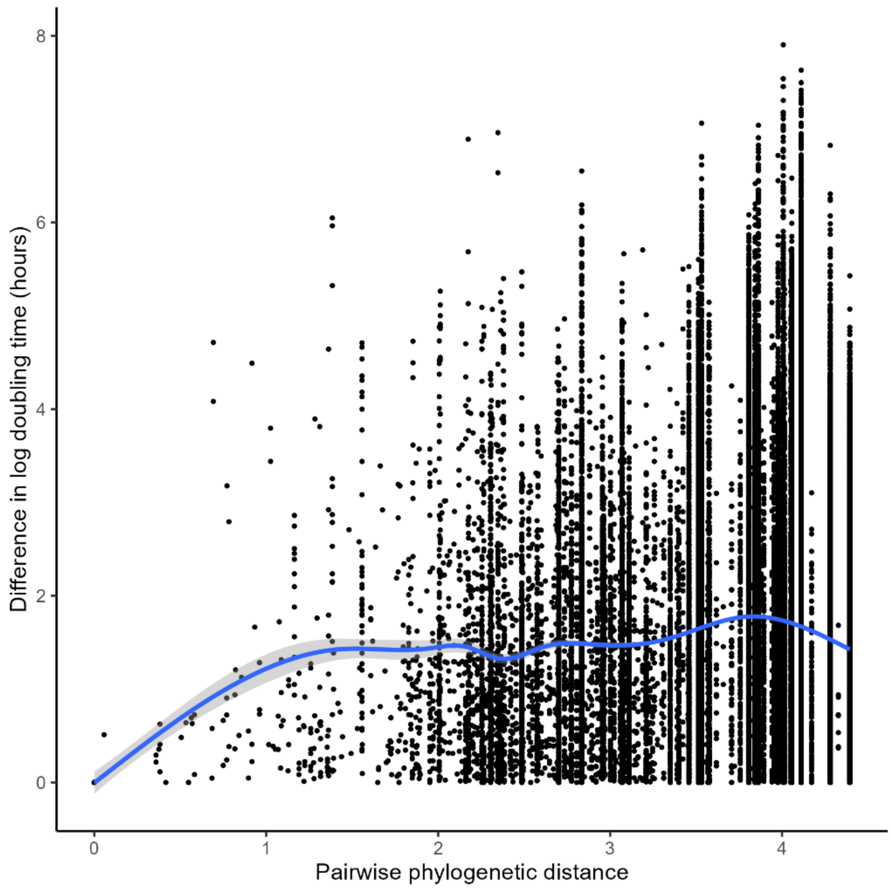

Figure S3. The pairwise phylogenetic distance vs. the difference in log-transformed doubling time between species. The blue line is the regressed line using a generalized additive model (GAM) with the default setting in the R ggplot2 function geom_smooth.

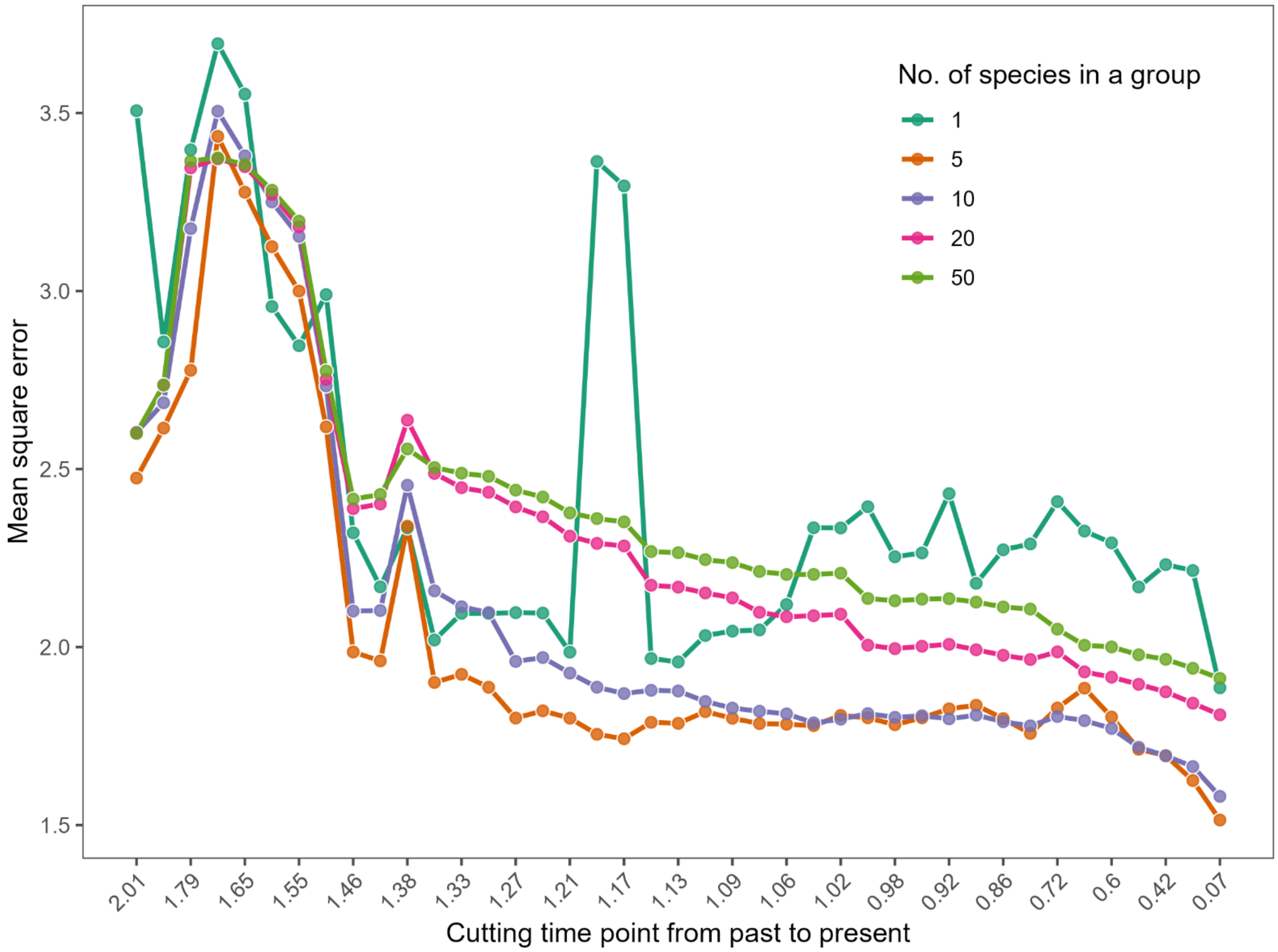

Figure S4. The MSE score of the NNM of different group sizes varies with the cutting time.

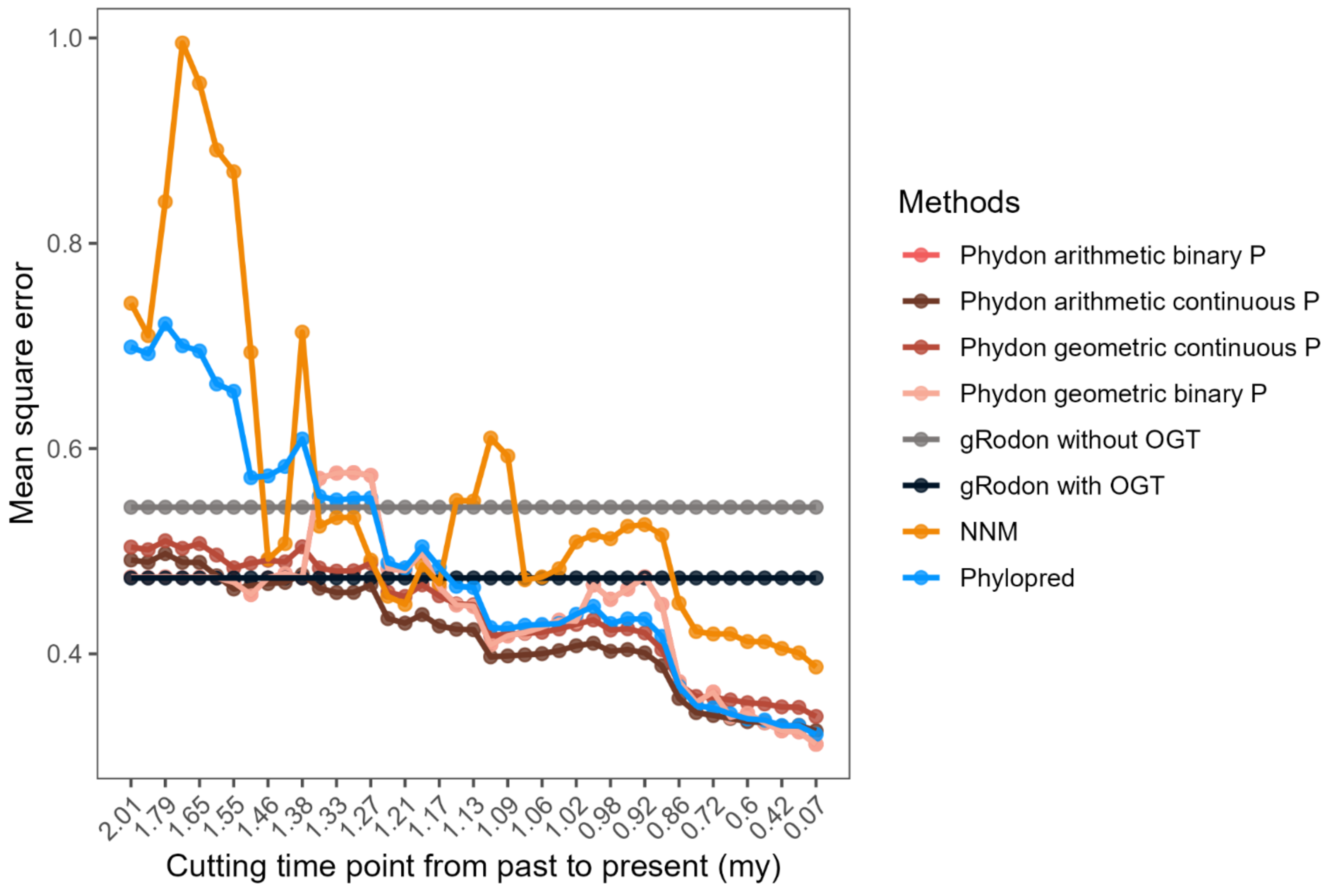

Figure S5. The prediction error of the seven models varies with different cutting time points between training and test data. Note that the arithmetic model and the geometric model with a binary *P* are identical. Thus, their MSE score lines overlap with each other.

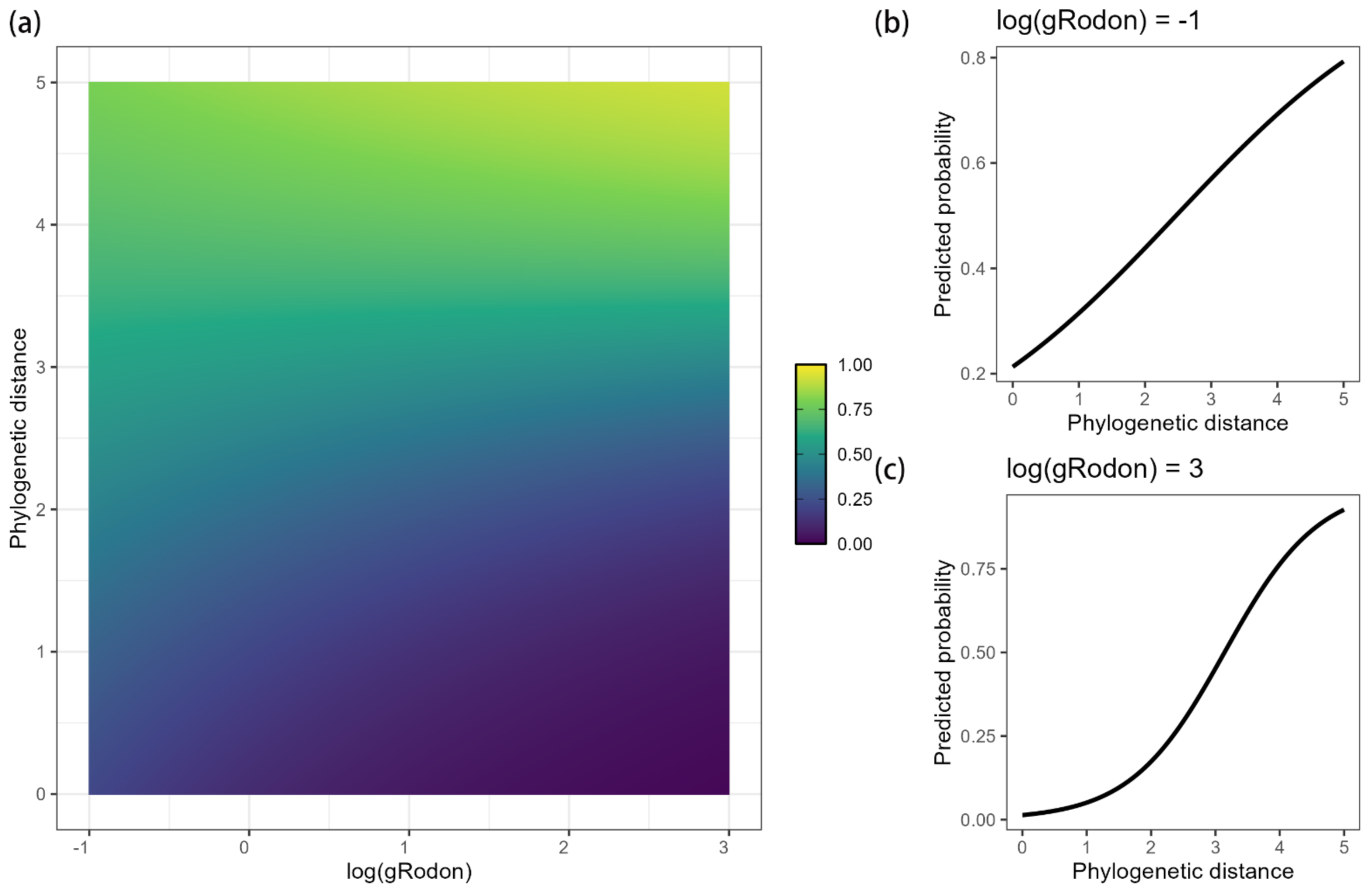

Figure S6. (a). The *P* values of the arithmetic model change with both the phylogenetic distance of the query genome to the training set and the expected growth rate of the query genome inferred by gRodon. (b), (c) the curve of *P* values at two extreme growth rates.

Supplementary References

1. Madin, J.S., et al., *A synthesis of bacterial and archaeal phenotypic trait data.* Scientific Data, 2020. **7**(1): p. 170.
